# Exuberant cutaneous lymphoepithelioma-like carcinoma: clinicopathological correlation in an aggressive case^⋆^^☆^

**DOI:** 10.1016/j.abd.2026.501412

**Published:** 2026-07-01

**Authors:** Alice Brandão Menezes Rocha, Airton Kenji Motizuki, Renata Mie Oyama Okajima, Josie Eiras Bisi dos Santos, Matheus Fonseca Barbosa

**Affiliations:** a Dermatology Outpatient Clinic Prof. Miguel Saraty de Oliveira, Centro de Ciências Biológicas e da Saúde II, Universidade do Estado do Pará, Belém, PA, Brazil; b Faculty of Medicine, Universidade Federal do Pará, Belém, PA, Brazil

Dear Editor,

Lymphoepithelial carcinomas (LECs) are rare and poorly differentiated malignant tumors, characterized by a typical histological pattern of intense reactive lymphoplasmacytic infiltrate associated with neoplastic epithelial cells.^1^, ^2^, ^3^ Although classically described in the nasopharynx, these tumors can arise in other organs, such as salivary glands (especially the parotid and submandibular), thymus, tonsils, and cervix. Primary or secondary cutaneous manifestation of this neoplasm is extremely uncommon and poorly documented in the literature.^1^, ^2^, ^3^

A 75-year-old male patient presented with a history of edema and pain in the right mandibular region for about a year, evolving with nodular lesions that showed progressive growth, extending to the anterior and posterior cervical region, and anterior and posterior thorax, without clinical evidence of nasopharyngeal involvement. He also reported weight loss, asthenia, and inappetence. On dermatological examination, erythematous-violaceous, infiltrated plaques and nodules with a shiny surface and irregular contours were observed in the mandibular, anterior and posterior cervical, and anterior and posterior thoracic regions, with associated cervical and axillary lymphadenopathy (Fig. 1A and 1B).

**Fig. 1:**
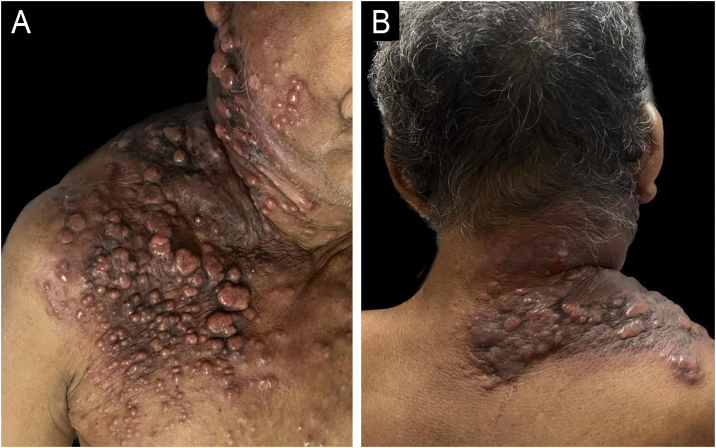
Infiltrated erythematous-violaceous plaque with a shiny surface extending from the mandibular region to the anterior and posterior right thorax.

A skin biopsy was performed, the histopathological examination of which revealed an invasive epithelioid malignant neoplasm associated with intense lymphocytic infiltrate (Fig. 2); subcutaneous cell tissue was not represented in the sample. Given these findings, the immunohistochemical analysis was indicated. This, in turn, demonstrated positivity for AE1/AE3, EMA, EP4 (focal), p40 (weak), in addition to negativity for CD45, RA, S100, CK7, CK20, and CD30, favoring the diagnosis of lymphoepithelioma-like carcinoma. It is noteworthy that CD45 showed positivity only in the reactive lymphocytic infiltrate, remaining negative in the epithelial neoplastic cells, a finding consistent with the lymphocytic predominance, characteristic of the infiltrate of this neoplasm (Fig. 3). Moreover, the sample showed diffuse positivity for EBER in the *in situ* hybridization method.

**Fig. 2:**
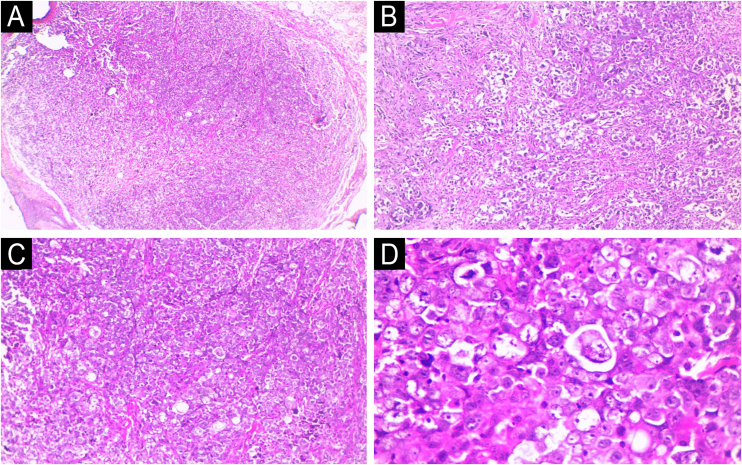
Histopathological characteristics. (A) Intradermal neoplasm with expansive growth, without connection to the epidermis and located mainly in the dermis. (B-C) Malignant epithelioid neoplasm, with intense, predominantly lymphocytic inflammatory infiltrate. (D) Malignant epithelial cells with pleomorphic nuclei, evident nucleoli and atypical mitoses (Hematoxylin & eosin; A: ×40, B–C: ×100, D: ×400).

**Fig. 3:**
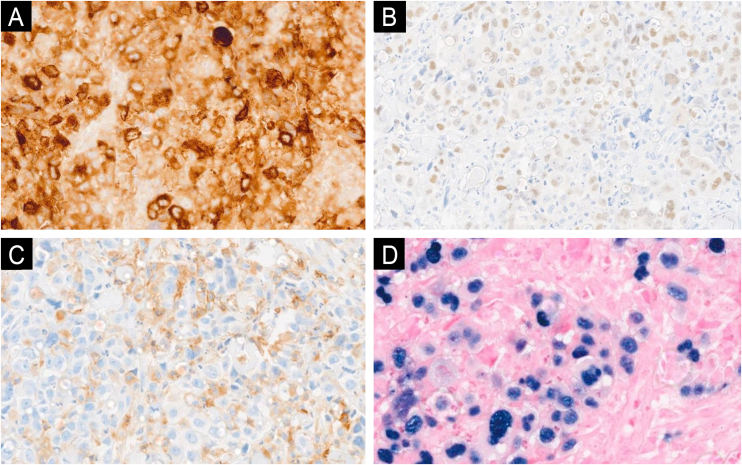
Immuno-histochemical analysis demonstrating positivity for EMA (A, ×400), p40 (B, ×400), CD45 (C, ×400) and EBER *in situ* hybridization (D, ×400).

Imaging tests were requested to investigate the primary site, and the patient was referred for urgent hospitalization, as he showed significant involvement of the general condition. However, he died less than a week after the initial care.

Lymphoepithelial carcinoma (LEC) occurs most frequently in the nasopharynx, although it has also been described in other anatomical sites, such as salivary glands and stomach. Cutaneous involvement is rare and poorly documented in the literature, generally being termed lymphoepithelioma-like carcinoma when located in extranasopharyngeal regions.^1^, ^2^, ^3^ When present, cutaneous manifestations generally affect the head and neck region, manifesting as erythematous nodules, plaques, or papules, with progressive evolution over months.^4^

The participation of Epstein-Barr virus (EBV) in the pathogenesis of cutaneous LEC is not yet fully established, with this association being better described in carcinomas originating from the upper airway.^2^, ^5^ From a histopathological point of view, there is a proliferation of malignant epithelial cells, with large, vesicular nuclei and evident nucleoli, immersed in a dense reactive inflammatory infiltrate, consisting predominantly of T-lymphocytes and, occasionally, plasma cells. This pattern may raise an initial diagnostic hypothesis of lymphoma, in addition to other differential diagnoses, such as follicular dendritic cell tumor and Merkel cell carcinoma.^2^ In this context, immunohistochemistry is crucial to confirm the epithelial origin of the neoplasm and exclude differential diagnoses, such as lymphomas (CD45 positive) and melanomas (S100 positive). In the present case, the positivity for AE1/AE3 and EMA, associated with the negativity for CD45 in the neoplastic cells, confirms the diagnosis of lymphoepithelial carcinoma.

The diffuse expression of EBER suggests an association with EBV and reinforces the possibility of an origin in the upper airway, since this relationship is classically described in nasopharyngeal lymphoepithelial carcinomas, being less frequent in primarily cutaneous cases. Moreover, the exuberant clinical presentation, associated with lymphadenomegaly and systemic involvement, strongly raises the hypothesis of metastatic disease. Although the histopathological examination did not show tumor cells inside the represented blood vessels, the sample obtained by biopsy corresponds to a limited area in relation to the clinical lesion extent, which restricts definitive conclusions regarding the tumor origin. No clinical evidence of nasopharyngeal involvement was identified at the time of evaluation; however, due to the patient's early death, it was not possible to complete the systemic investigation by imaging methods, constituting a relevant limitation of the case and preventing the unequivocal determination of the primary neoplasm site.

Treatment of lymphoepithelial carcinoma mainly involves wide surgical resection, which may be combined with radiotherapy in cases of recurrence or lymph node metastases.^4^ Considering its wide clinical variability and rarity of cutaneous manifestations, the importance of histopathological examination and immunohistochemical study for diagnostic confirmation is highlighted. Early request of these tests by the dermatologist is essential to reduce diagnostic delays and allow appropriate therapeutic interventions, minimizing the risk of unfavorable outcomes.

## ORCID ID

Renata Mie Oyama Okajima: 0000-0003-2168-5515

Josie Eiras Bisi dos Santos: 0000-0001-8512-3920

Matheus Fonseca Barbosa: 0009-0009-8114-5159

## Financial support

None declared.

## Authors' contributions

Alice Brandão Menezes Rocha: Drafting and editing of the manuscript or critical review of important intellectual content; collection, analysis and interpretation of data; intellectual participation in the propaedeutic and/or therapeutic conduct of the studied cases; critical review of the literature; approval of the final version of the manuscript.

Airton Kenji Motizuki: Drafting and editing of the manuscript or critical review of important intellectual content; collection, analysis and interpretation of data; critical review of the literature; approval of the final version of the manuscript.

Renata Mie Oyama Okajima: Drafting and editing of the manuscript or critical review of important intellectual content; collection, analysis and interpretation of data; effective participation in research orientation; intellectual participation in the propaedeutic and/or therapeutic conduct of the studied cases; critical review of the literature; approval of the final version of the manuscript.

Josie Eiras Bisi dos Santos: Drafting and editing of the manuscript or critical review of important intellectual content; collection, analysis and interpretation of data; effective participation in research orientation; intellectual participation in the propaedeutic and/or therapeutic conduct of the studied cases; critical review of the literature; approval of the final version of the manuscript.

Matheus Fonseca Barbosa: Drafting and editing of the manuscript or critical review of important intellectual content; collection, analysis and interpretation of data; approval of the final version of the manuscript.

## Research data availability

Does not apply.

## Conflicts of interest

None declared.
